# Erratum

**DOI:** 10.1590/1518-8345.0000.2824

**Published:** 2016-07-18

Regarding the article "A multiprofessional perspective on the principal barriers to universal health coverage and universal access to health in extremely poor territories: the contributions of nursing", with DOI number: 10.1590/1518-8345.1042.2688, published in the Rev. Latino-Am. Enfermagem. 2016;24:e2688, page 1:

Where was written:

"Rev. Latino-Am. Enfermagem. 2016;24:e2688"

Now Read:

"Rev. Latino-Am. Enfermagem. 2016;24:e2795"

Regarding the article "Access the Unified Health System actions and services from the perspective of judicialization", with DOI number: 10.1590/1518-8345.1012.2689, published in the Rev. Latino-Am. Enfermagem. 2016;24:e2689, page 1:

Where was written:

"Rev. Latino-Am. Enfermagem. 2016;24:e2689"

Now Read:

"Rev. Latino-Am. Enfermagem. 2016;24:e2797"

Regarding the article "Health-related quality of life as a predictor of mortality in patients on peritoneal dialysis", with DOI number: 10.1590/1518-8345.0786.2687, published in the Rev. Latino-Am. Enfermagem. 2016;24:e2687, page 1:

Where was written:

"Rev. Latino-Am. Enfermagem. 2016;24:e2687"

Now Read:

"Rev. Latino-Am. Enfermagem. 2016;24:e2794"

